# Spectrophotometric Method for Analysis of Metformin Hydrochloride

**DOI:** 10.4103/0250-474X.51947

**Published:** 2009

**Authors:** G. Mubeen, Khalikha Noor

**Affiliations:** Al-Ameen College of Pharmacy, Hosur Road, Bangalore-560 027, India

**Keywords:** Metformin hydrochloride, ninhydrin

## Abstract

A simple and sensitive spectrophotometric method has been developed and validated for the estimation of metformin hydrochloride in bulk and in tablet formulation. The primary amino group of metformin hydrochloride reacts with ninhydrin in alkaline medium to form a violet colour chromogen, which is determined spectrophotometrically at 570 nm. It obeyed Beer's law in the range of 8-18 μg/ml. Percentage recovery of the drug for the proposed method ranged from 97-100% indicating no interference of the tablet excipients. The proposed method was found to be accurate and precise for routine estimation of metformin hydrochloride in bulk and from tablet dosage forms.

Metformin hydrochloride, chemically 1, 1-dimethylbiguanide hydrochloride[1] (C_4_H_11_N_5_.HCl) is white crystalline powder, hygroscopic and freely soluble in water, used as a hypoglycemic drug[2]. Literature survey reveals that only few methods like HPLC and GC have been reported for estimation of the metformin hydrochloride in pharmaceutical formulations and biological fluids[3–9]. Official method includes UV spectrophotometric method for estimation of the drug from the tablets[1]. However no colorimetric methods are reported for estimation of metformin hydrochloride in bulk and in formulations.

The present work describes a new simple spectrophotometric method based on the reaction between amino group of metformin hydrochloride with ninhydrin to form a violet colored complex, which shows absorption maxima at 570 nm.

The reference standard of metformin hydrochloride was procured as gift sample from Micro Labs, Bangalore and tablets (Obimet 500 mg, Kare Labs Pvt. Ltd. Goa) were utilized for the study. Ninhydrin and all other chemicals, solvents utilized were of AR grade. A double beam spectrophotometer (Shimadzu-UV-1601) was employed for measurement of absorbance.

A standard solution of metformin hydrochloride was prepared by dissolving 100 mg of the drug in 100 ml of distilled water and further diluted with water to get concentration of 100 μg/ml. Twenty tablets were weighed, powdered and the powder equivalent to 100 mg of metformin hydrochloride was accurately weighed, dissolved in 100 ml of distilled water, filtered through Whatmann filter paper No: 41 and diluted further to get a concentration of 100 μg/ml.

To a series of (S_1_, S_2_, S_3_, S_4_, S_5_) 25 ml volumetric flasks, aliquots of 2.0 to 4.5 ml of the standard solution of metformin hydrochloride, 1.5 ml of 5M NaOH, 2.2 ml of 1% ninhydrin solution and 10 ml of water was added, heated on a water bath for 30 min, cooled and volume adjusted to 25 ml with water and the absorbance of the solution in each flask was measured at 570 nm against reagent blank. The absorbance of sample solution was also measured and the amount of metformin hydrochloride present in tablet formulation was determined by extrapolating from the calibration curve. The results are shown in the Table 1. In order to ascertain the suitability and reproducibility of the proposed method, recovery studies were carried out by adding known quantities of standard metformin hydrochloride to the previously analyzed sample and the mixtures were reanalyzed by the proposed method. The results are shown in the Table 2. The percentage recovery of metformin hydrochloride was found in the range of 97-100% indicating that there is no interference by the excipients in the method.

**TABLE 1:** ANALYSIS OF METOFRMIN HYDROCHLORIDE IN TABLETS

Formulation (Tablets)	Labelled amount (mg)	Amount found (mg)	% Recovery by proposed method
Obimet	500	485.41*	97.08
Obimet	500	493.57*	98.71
Obimet	500	494.37*	98.87

Table showing analysis of metformin hydrochloride in tablets by proposed spectrophotometric method.

* Average of three determinations

**TABLE 2:** RECOVERY STUDIES OF METFORMIN HYDROCHLORIDE

Volume of Standard (ml)	Volume of sample (ml)	Concentration of sample (μg/ml)	Absorbance* at 570 nm	From standard graph (μg/ml)	Recovery of standard	% recovery ±SD
2.5	1.0	04	0.080	14.00	10.00	100.0±0.99
3.0	1.0	04	0.091	15.82	11.82	98.50±0.98
3.5	1.0	04	0.099	17.64	13.64	97.42±0.97

Table showing % recovery of metformin hydrochloride by the proposed method.

* Average of three determination

The proposed method is simple, accurate, precise sensitive and can be successfully applied for routine quantitative estimation of metformin hydrochloride in bulk and solid dosage forms. The summary of the method developed is shown in the Table 3.

**TABLE 3:** SUMMARY OF METHOD DEVELOPED

Parameter	Results
Absorption maxima (λ max)	570 nm
Stability of color	15 min
Beers law range	8-18 μg/ml
Sandell's sensitivity	0.17543 μg/cm^2^/0.001 absorbanc units
Molar absorptivity	5.7 × 10 mole−1 cm−1

Table showing summary of analytical parameters for developed spectrophotometric method
